# Supplementary material for: Evaluating systematic targeted universal testing for tuberculosis in primary care clinics of South Africa: A cluster-randomized trial (The TUTT Trial)
Source: PLoS Med. 2023 May 22;20(5):e1004237. doi: 10.1371/journal.pmed.1004237 (PMC10263318; doi:10.1371/journal.pmed.1004237)
Supplement: S1 Table — (DOCX) [file pmed.1004237.s002.docx]

| Supplementary Table 1: Characteristics and yield of sputum TB testing in participants recruited in intervention clinics in the cluster randomized trial. | | | | |
| --- | --- | --- | --- | --- |
| Baseline characteristics and yield | **Overall** | **Xpert positive, trace included** | **Xpert positive, trace excluded** | **Xpert and/or culture positive, trace excluded** |
| Number of participants, (# [%]) | 30513 | 2327 (7.6%) | 1552 (5.1%) | 1823 (6.0%) |
| Age (yr.) (median [IQR]) | 37 (30 - 46) | 35 (28 - 44) | 35 (27.5 - 43) | 35 (28 - 43) |
| 18-19 | 694 | 67 (9.7%) | 54 (7.8%) | 62 (8.9%) |
| 20-29 | 6830 | 605 (8.9%) | 437 (6.4%) | 495 (7.2%) |
| 30-39 | 10020 | 848 (8.5%) | 544 (5.4%) | 640 (6.4%) |
| 40-49 | 7132 | 485 (6.8%) | 314 (4.4%) | 376 (5.3%) |
| 50-59 | 3937 | 225 (5.7%) | 142 (3.6%) | 177 (4.5%) |
| 60+ | 1897 | 97 (5.1%) | 61 (3.2%) | 73 (3.8%) |
| Female (# [%]) | 18934 | 1120 (5.9%) | 639 (3.4%) | 769 (4.1%) |
| Male (# [%]) | 11553 | 1205 (10.4%) | 912 (7.9%) | 1052 (9.1%) |
| Asymptomatic (# [%]) | 22255 | 1350 (6.1%) | 812 (3.7%) | 994 (4.5%) |
| Symptomatic (# [%]) | 8217 | 974 (11.9%) | 737 (9.0%) | 826 (10.1%) |
| HIV seronegative (# [%]) | 8192 | 780 (9.5%) | 591 (7.2%) | 665 (8.1%) |
| HIV status unknown (no [%[) | 587 | 71 (12.1%) | 63 (10.7%) | 64 (10.9%) |
| People living with HIV (# [%]) | 21734 | 1476 (6.8%) | 898 (4.1%) | 1094 (5.0% |
| CD4 count known† (#) | 8665 | 504 | 251 | 330 |
| CD4 count (cells/mm^3^) (median [IQR]) | 422 (248 - 613) | 378 (219 - 591.5) | 336 | 352 (197 - 558) |
| ART status known† (#) | 8510 | 655 | 348 | 424 |
| On ART† (# [%]) | 7421 | 479 (6.5%) | 238 (3.2%) | 294 (4.0%) |
| Not on ART† (# [%]) | 1230 | 197 (16.0%) | 130 (10.6%) | 150 (12.2%) |
| TB contact (# [%]) | 12492 | 1131 (9.1%) | 821 (6.6%) | 936 (7.5%) |
| Prior TB (# [%]) | 1573 | 242 (15.4%) | 171 (10.9%) | 188 (12.0%) |
| Province |  |  |  |  |
| Gauteng (# [%]) | 6593 | 156 (2.4%) | 102 (1.5%) | 131 (2.0%) |
| Kwazulu-Natal (# [%]) | 14381 | 1118 (7.8%) | 906 (6.3%) | 1027 (7.1%) |
| Western Cape (# [%]) | 9539 | 1053 (11.0%) | 544 (5.7%) | 665 (7%) |

Abbreviations: ART, antiretroviral therapy; HIV, human immunodeficiency virus; IQR, interquartile range; TB, tuberculosis.

†The protocol was amended to include these questions partway through the study, and not all participants have this data.
