# Supplementary figures and images for: Evaluating systematic targeted universal testing for tuberculosis in primary care clinics of South Africa: A cluster-randomized trial (The TUTT Trial)
Source: PLoS Med. 2023 May 22;20(5):e1004237. doi: 10.1371/journal.pmed.1004237 (PMC10263318; doi:10.1371/journal.pmed.1004237)

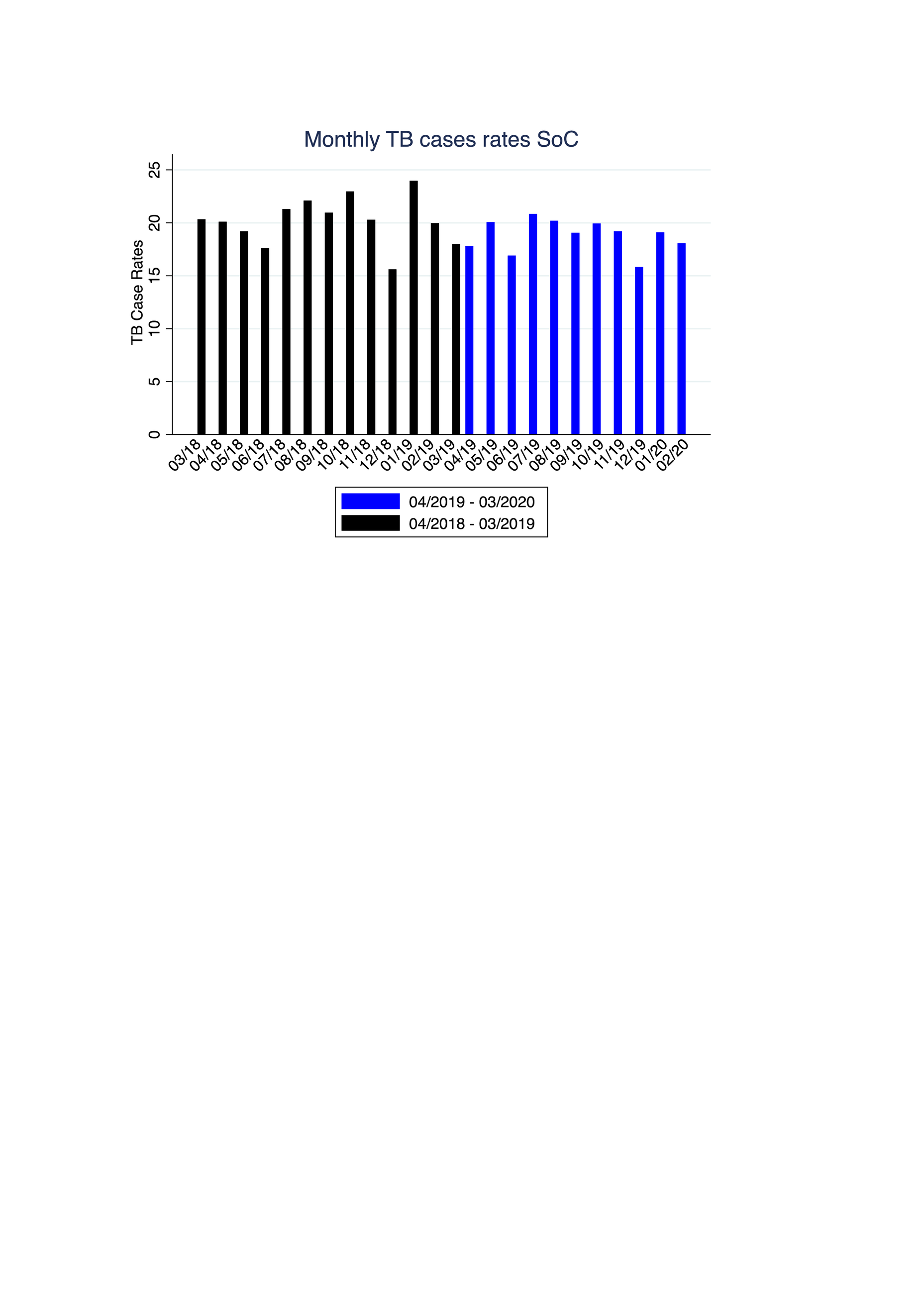

Supplement: S1 Fig — (TIF) [file pmed.1004237.s003.tif]

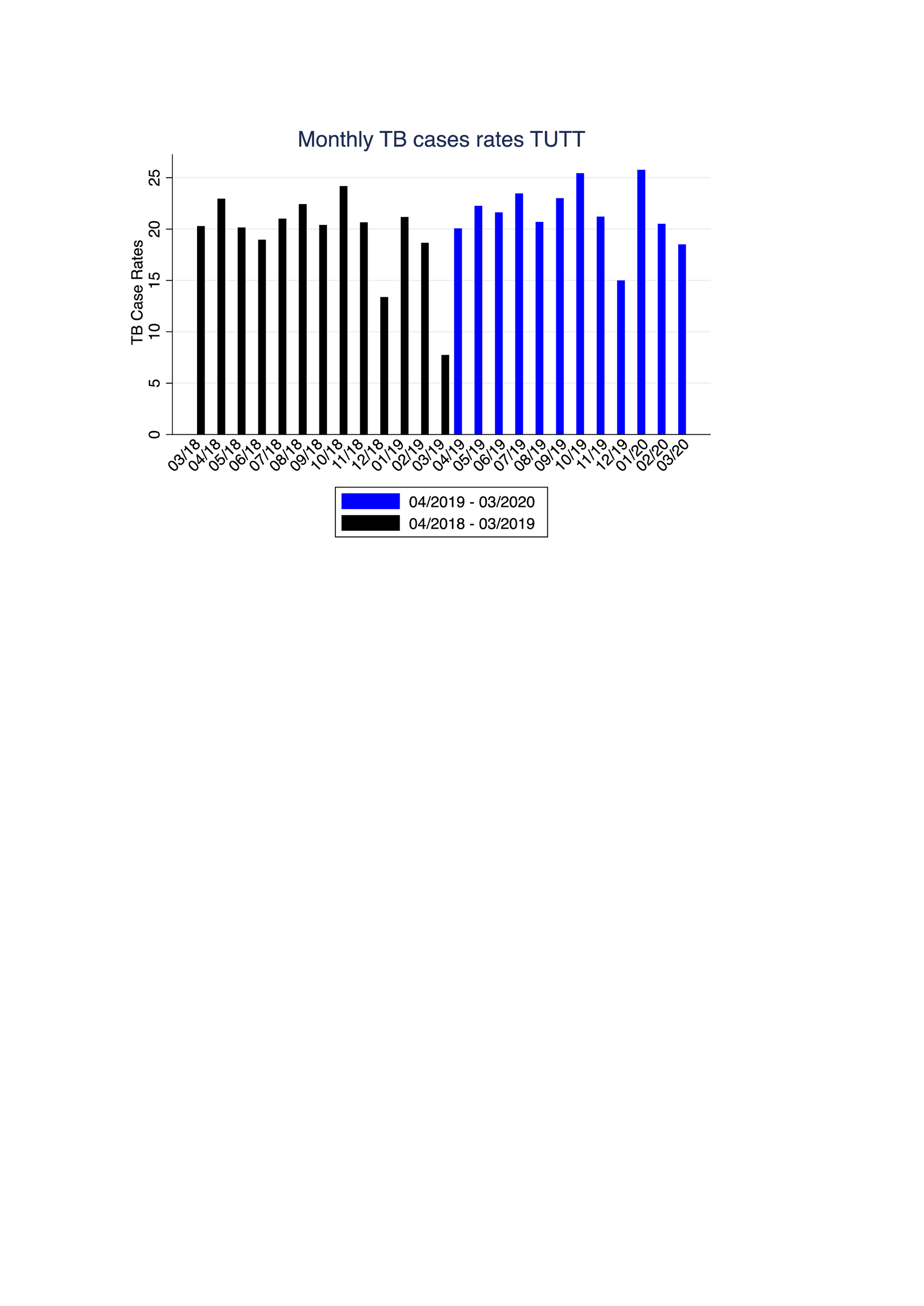

Supplement: S2 Fig — (TIF) [file pmed.1004237.s004.tif]
