# Supplementary material for: Evaluating systematic targeted universal testing for tuberculosis in primary care clinics of South Africa: A cluster-randomized trial (The TUTT Trial)
Source: PLoS Med. 2023 May 22;20(5):e1004237. doi: 10.1371/journal.pmed.1004237 (PMC10263318; doi:10.1371/journal.pmed.1004237)
Supplement: S1 Information — (PDF) [file pmed.1004237.s005.pdf]

STRATEGIC INNOVATION DEMONSTRATION STUDIES: TARGETED UNIVERSAL TESTING FOR TB (TUTT)

Version 4.0 dated 10 March 2020

Principal Investigator

Perinatal HIV Research Unit (PHRU)

Dr Neil Martinson

Investigators

Dr. Limakatso Lebina (Perinatal HIV Research Unit)

Ms. Katlego Motlhaoleng (Perinatal HIV Research Unit)

Ms. Minja Milovanovic (Perinatal HIV Research Unit)

Dr. Harry Hausler (TBHIVCare)

Dr. Sipho Nyathi (Aquity Innovations)

Dr. Lucy Connell (Right to Care)

Dr. Marianne Black (NHLS)

Dr. Keeren Lutchiminarain (NHLS)

Dr. Khine Swe Swe Han (NHLS)

Dr. Natalie Beylis (NHLS)

Dr. Rebecca Berhanu (Boston University /HERO)

Dr Anthony Kinghorn (PHRU)

Background

Though effective TB vaccines or new therapeutic drugs are likely necessary to eliminate TB, it has been suggested that "**doing the basics better**" with existing technologies and diagnostics could lead to a significant reduction in morbidity, mortality and transmission of TB. We posit that by improving the case detection rate, and loss to follow up between diagnosis of TB and TB treatment initiation, health services could have a marked impact on reducing the burden of TB in South Africa (1, 2).

Diagnostic gaps in a global and local context

The most recent World Health Organization (WHO) Global TB Report suggests that 4.1 million cases worldwide in 2016, representing 39% of the estimated incident cases in the year, were "missing" or not notified as starting TB therapy (3), suggesting large numbers of undiagnosed cases. South Africa (SA) is one of the top ten countries that contributes to the total missing patients (3). A recently published analysis of the cascade of linkages to TB care in SA estimated that in 2013, approximately 5% of TB cases did not access diagnostic tests, and 13% were missed during diagnosis by testing false-negative (1). Undetected TB leads to both increased transmission at a community level, as well as significant morbidity and mortality at an individual level, the latter well demonstrated by multiple autopsy studies. Our own recent findings from the North West Province found evidence of pulmonary TB in close to a third of participants who died at home from natural causes, with no TB previously being diagnosed or treated(4). Another study in KwaZulu-Natal showed approximately half of sampled adult cadavers of patients dying at a district hospital were culture-positive for TB, including those already on TB treatment and those who were not considered as having TB prior to death (5).

Active case-finding in SA

Given the problem of the diagnostic gap, global and national guidelines advocate active case-finding through screening activities within certain sub-populations at particular risk of TB (6, 7). Passive case-finding alone is insufficient at identifying the majority of TB cases, and WHO policy recommendations strongly suggest active case finding in, among others: people living with HIV (PLHIV), miners, prisoners, health care workers, household TB contacts, those with diabetes mellitus and those with recurrent TB (8). However, current active case finding relies on symptom-screening for all people falling within high risk groups, using the WHO-recommended screening tool for: persistent cough (or cough of any duration if HIV positive), persistent fever, drenching night sweats, and unexplained loss of weight (7). If any of these symptoms are present, a sputum test, ideally a Xpert MTB-RIF (Cepheid) if available, is performed (7). The symptom-screening tool was developed based on several studies included in a meta-analysis, and designed to maximize sensitivity and negative predictive value, and is therefore understood to be most useful at excluding individuals in whom TB is unlikely (9). There are however, studies that report lower sensitivity to the screening tool, including in PLHIV and our own studies in HIV positive pregnant women (10-12). Indeed, subsequent data from an implementation science study conducted by our group - a cluster randomized trial where primary healthcare clinics providing prenatal care were randomized to provide either universal sputum testing for TB, or to standard of care (SoC) where symptom-based testing was provided - showed a tenfold reduction in case detection using a universal testing approach (3.6% of HIV-infected pregnant women in the universal testing arm were diagnosed

with laboratory confirmed TB compared to 0.36% in the symptom-based testing Primary healthcare (PHC) clinics). The study initially used Xpert MTB/RIF as the only assay but approximately half way through the study, sputa were split: half for Xpert MTB/RIF and half for liquid mycobacterial culture (MGIT) – MGIT detected almost fourfold the rate of Xpert MTB/RIF in universal testing clinics where all women provided a sputum. In the SoC clinics, the study employed a counsellor to specifically ask each woman about her symptoms and to ensure that a sputum was taken if symptoms were reported. Fidelity to protocol was high with over 95% of women in the universal based testing clinics having a sputum test result. In South Africa, all clinic attendees are required to have a TB symptom screen at entry, however, there is some concern the large scale at which symptom-screening takes place in PHC clinics in SA negatively impacts the quality of symptom-screening that takes places (13). Recent data from PHC clinics in the East Rand of Johannesburg suggests that despite a dramatic increase in the number of people who were symptom screened, there was no increase in total number of TB cases diagnosed.

High risk groups

The highest risk group for developing TB is HIV-infected individuals, who are at approximately tenfold risk to develop TB than HIV seronegative individuals (14). South Africa has an extreme burden of HIV-TB co-infection, and efforts to meaningfully integrate HIV and TB services are well-established. Marked success has been achieved at ensuring that diagnosed TB cases are also assessed for HIV, with 96% of notified TB cases also having a documented HIV status in 2016, and 88% of those who are infected also being on antiretroviral therapy (3). However, many HIV-infected individuals have undiagnosed TB. It has been suggested that especially in newly HIV diagnosed, the prevalence of TB is so high that universal TB testing should take place with all such individuals (15). Pregnant women with HIV are at particularly high risk, with HIV and TB co-infection currently being the leading cause of death in women of reproductive age and of maternal mortality in South Africa (16, 17).

Household contacts of TB cases have long been established as at high risk for TB and are a target for screening programs, particularly in the case of smear positive and cavitatory TB disease (18-20). Our own data has confirmed this and additionally shown culture-confirmed TB can be detected early in contacts prior to the development of symptoms (18). Those with a history of previous TB are also at greater risk, especially in the context of HIV co-infection (21).

Clearly there are other groups at high risk for TB, including miners and, health care workers (6). Similarly, diabetes mellitus, tobacco smoking and heavy alcohol use are independent risk factors for TB disease, with 8% of TB cases worldwide attributable to smoking (22, 23). Importantly, these risk factors are not just individual but are also compounding in combination. A meta-analysis of a large set of patients across multiple high-burden TB countries found that a combination of smoking, alcohol use, low BMI and diabetes increased the risk of TB symptoms by three to four fold compared to the general population (27). Recently, several attempts have been made to improve this screening process by risk stratification, including several studies that developed clinical scoring systems to identify PLHIV who are most likely to also have TB. These studies were designed to streamline the TB

screening process in the context of HIV care, to reduce the number of unnecessary investigations for TB in a resource-scarce setting, and to prioritize those with a high likelihood of having TB. The final clinical score developed from the XPHACTOR study included: being pre-ART or on ART for less than 3 months as high scoring, as well as having a CD4 of less than 350 (for a moderate score) or less than 200 (for a high score) and a body mass index (BMI) less than 18.5 for a high score (15, 28). This study was not specifically designed to detect more cases of TB, but rather to reduce the number of sputum tests ordered.

Our intention, however, is to find more cases of TB, not to reduce the number of tests. We are concerned that adding additional clinical criteria that may not be immediately available at the time of identifying targeted groups, may reduce the number that are investigated. To reduce implementation complexity, our plan is to keep the number of targeted groups to four or fewer.

South Africa currently uses Xpert Ultra (Cepheid, Sunnyvale CA) for diagnosis of TB, a molecular assay with high sensitivity and specificity. There is little evidence on the superiority of Xpert Ultra as a first-line screening test since early studies establishing test characteristics for this and the previous model (Xpert MTB/RIF) included only presumptive TB cases. Results of several randomized controlled trials (RCTs) have however found that Xpert tests increase rates of treatment initiation and shortens time to TB treatment (29, 30). The new version of this assay, the Xpert MTB/RIF Ultra has been developed and recently determined to have better test performance in smear negative TB and HIV-positive TB patients. (14) There is no data that validates the use of the Ultra in urine samples but the STAMP trial Xpert MTB/RIF has been shown to detect substantially more cases of TB in hospitalized patients. Liquid mycobacterial culture, using the Mycobacterial Growth in Tube (MGIT) platform (Becton Dickinson, Franklin Lakes, NJ), remains the gold standard of TB diagnosis; though turnaround time to results is in the order of weeks, and for early paucibacciliary TB, it may take up to 42 days for a positive culture result (31).

Performance of Xpert Ultra for active case finding

Although Xpert Ultra showed greater sensitivity for TB detection in smear-negative disease and in people with HIV in recent trials, this increased sensitivity came at the cost of lost specificity. The clinical significance of Ultra positive, culture-negative testing remains unclear despite the widespread use of the test in South Africa and globally since 2017. False positive have been associated with the lowest semi-quantitative category of TB detected by Ultra which is called ‘trace’ and with a past history of TB. The performance of Xpert Ultra in active case finding trials has also not yet been described in the literature.

Contact Tracing

Contact tracing cards (CTC) are handed to patients as a means of communicating with people that they may have infected. In TB, CTC have been used as a method of connecting with TB patients and passively getting a contact to be screened for TB. The use of CTC was reported to be particularly useful in a previous South African study in Johannesburg (35): 26% of contacts of index cases receiving CTCs reported for TB testing of whom 12% were diagnosed with TB; 68% of contacts reported to clinics within 2 weeks of receiving the CTC and 98% of

participants reported that CTC was acceptable. However, only symptomatic contacts were screened for TB in that study. In an observational study from Malawi, passive contact tracing (essentially requesting index cases to tell their contacts to come to the clinic) was inferior to active case finding in detecting TB in contacts (36); merely requesting contacts to come for screening returned over 5 contacts per TB case to the clinic, almost identical to active case finding, which required a household visit. Clearly the CTC strategy requires fewer costly inputs than household visit-based contact tracing.

mHealth mobile interventions

Access to mobile phones has substantially increased in South Africa. t is said that in several years only smart phones will be supported in South Africa ensuring a rapid move away from the older feature phones. This has created opportunities for the integration of mobile phone technology as a health intervention tool. Within the field of HIV, mobile Health (mHealth) applications have been explored extensively including: prevention, diagnosis, data collection, treatment, adherence, monitoring and surveillance (37). Mobile text messages, are an innovative approach (38) that have the potential to promote and raise awareness on health and disease and can be used by healthcare providers to communicate better with patients and contacts in a cost-effective manner. In 2017 the World Health Organization (WHO) released guidelines on the use of mobile technology for TB treatment adherence. Furthermore, a study conducted by Khan et al in Pakistan used mobile phones, in combination with other intervention, for case finding and reported an increased case detection rate of 2.21 times (39). However, mobile health interventions remain underutilized within tuberculosis contact tracing. We plan to invite all TB contacts for TB testing. They may have potential to reach contacts and encourage them to attend clinics thereby increasing yield or case detection rates. Our study will be the first that will assess the effectiveness of a targeted mHealth intervention compared to standard contact tracing in Sub-Saharan Africa in a randomized fashion.

Objectives

By conducting a factorial cluster randomized trial of health faculties, our overall objective is to measure the impact of implementing two innovative strategies that could diagnose at least 25% more cases of TB among health facility attendees, then current symptom-based testing practice.

Specific aims

1. Our primary aim is to compare whether **targeted universal testing of high risk groups for TB** (TUTT) using MGIT and Xpert Ultra detects more cases than standard of care (SoC) which is symptom-based testing for all attendees (Strategy 1). Hypothesis: TUTT will detect 25% more cases than SoC.

2. An exploratory aim is to determine whether **a mHealth intervention is better than standard contact tracing** in increasing contacts screened and total TB cases detected. (Strategy 2) Hypothesis: mHealth will lead to additional TB cases than standard contact tracing.

3. To determine **costs and incremental cost effectiveness** of the two strategies.
4. **Define test performance of Xpert Ultra as a screening tool for TB diagnosis when used for active case finding when compared to liquid culture as a gold standard**

Outcomes

All outcomes (apart from cost) will be abstracted from routine PHC clinic data and compared between primary randomization arms.

Comparisons between TUTT and SoC facilities:

1. **Numbers of cases of TB diagnosed over the entire duration of the study**. We will additionally compare the total number of TB cases diagnosed with historical data, by facility: in the 12 months prior to the intervention to that from the 2nd - 12th month of the intervention; and if required match holiday seasons between time periods (Easter, end of year including Christmas and New Year's holidays which are quiet periods).
2. **Number tested to diagnose one TB case**. We will also report the number TB tested to diagnose one case in the high risk and the moderate risk groups in the TUTT PHC clinics.
3. **Number of new TB cases notified and initiated on treatment**.

Comparisons between mHealth and standard contact tracing PHC clinics.

4. **Numbers of additional cases of TB diagnosed** over the entire duration of the study.
5. The number of **contacts screened**.
6. The **incremental yield** of mHealth comparing mHealth+TUTT vs standard contact tracing + TUTT PHC clinics
7. We will report **incremental cost and cost effectiveness** for each of the interventions.

Test performance of Xpert Ultra as a screening tool for TB diagnosis when used for active case finding

8. Describe sensitivity, specificity, positive and negative predictive value of the Ultra test overall and in the three TUTT test populations: 1) HIV infected; 2) history of TB in the preceding 2 years; and 3) close contact with a person with TB. Mycobacteria Growth Indicator Tube (MGIT) liquid culture will be considered as the gold standard test in this analysis.
9. Examine the relationship between HIV status, CD4 count, anti-retroviral therapy (ART) initiation, viral load suppression, and prior history of TB (within 1 year, and within 2 years) on the risk of Ultra-trace and culture discordance as compared to individuals who are Ultra-trace and culture positive

Methodology

Setting

South Africa is divided into 9 provinces, with 52 health districts, 3198 PHC clinics, and 253 district hospitals. The study will be conducted in PHC clinics and district hospitals in three provinces in South Africa. Gauteng (GP), Kwa-Zulu Natal (KZN) and Western Cape (WC) have five, eleven and six health districts with 337, 595 and 210 PHC clinics, respectively. Each province also has at least two district hospitals. These provinces along with the Eastern Cape have the highest TB burden in South Africa. TB incidence rates for GP, KZN and WC in 2015 were 330, 685 and 681 per 100 000, respectively (36). At the district level eThekwini (KZN), Cape Town (WC), and Johannesburg (GP) had the highest TB burden in 2015 with 24 588, 23 815 and 15 912 cases respectively. Tuberculosis patients in Gauteng and KwaZulu-Natal had the highest HIV co-infection rates at 68.4% and 63.6%, respectively.

Study design

A three-province, cluster-randomized trial will be conducted to evaluate two interventions that could diagnose more TB patients in the facilities randomized to receive either intervention, both interventions or neither.

Firstly, we will compare targeted universal testing for TB (TUTT) to the current standard of care (SoC) to identify TB cases. We will select 60 facilities approximately equally from the three high burden provinces (WC, GP and KZN), and will randomise 60 PHC clinics across the three provinces to one of the two study arms (TUTT vs SoC). Secondly, we will assess the benefits of integrated mHealth contact tracing and standard contact tracing, which involves distributing contact tracing slips (CTS) to TB cases by independently randomizing - a second randomisation process of all 30 PHC clinics to receive either the integrated mHealth vs standard contact tracing. By so doing we will have three groups of PHC clinics and be able to determine the independent, and joint impact of TUTT and SoC on increasing the total number of TB patients diagnosed and compare the sub-arms (mHealth and standard contact tracing) amongst each other and SoC.

Facility Eligibility Criteria

Only PHC clinics that have diagnosed more than 20 cases of TB per month in 2016 will be eligible to participate in this study. Facilities will be identified using the National Health Laboratory Service's (NHLS) National Priority Programme Central Database which receives regular transmissions from every Xpert TB diagnostic machine in the public sector. Using this data, we are able to assess, from each facility, how many specimens were tested, and the yield of TB. Professors Wendy Stevens and Lesley Scott have already agreed to provide this data and did a preliminary assessment of the facilities served by Xpert machines for this proposal (Table 1). PHC clinics will not be conducting any other TB research for the duration of the study and for one year prior to the study to ensure no interference with study effect. Study investigators will decide if there is a possible overlap of studies whether the PHC clinic should be excluded from randomization.

Table 1: Population of PHC clinics from which our sample will be drawn. Number of health facilities diagnosing at least 15 cases of TB per month by province.		
	Number of facilities diagnosing >15 TB cases per month	Number of facilities diagnosing >20 TB cases per month
KwaZulu-Natal	76	46
Western Cape	56	31
Gauteng	33	22

Each selected PHC clinic will be randomized to either the TUTT or SoC. Based on the number of facilities diagnosing ≥20 TB cases per month, we will include 24 clinics from KwaZulu-Natal, 24 in Western Cape and 12 in Gauteng. We will ensure that the selected facilities are sufficiently far apart from each other, and not on the same major transport or referral routes, to avoid contamination between arms. Moreover, we will avoid large teaching and referral hospitals and district hospitals.

Study procedures

Targeted Universal TB Testing Facilities

In the TUTT arm, all patients attending the PHC clinic will be categorized as high or moderate risk for TB disease according to pre-defined factors. High-risk groups will have sputum collected for World Health Organization approved TB assay the Xpert Ultra, and MGIT testing regardless of the presence of TB symptoms. For those whom the Xpert result is positive, a second sputum sample will be collected to validate a true positive Xpert result. Xpert Ultra is due to be implemented across South Africa in early 2018.

High-risk groups:

- **All HIV-infected individuals older than 18**. Although there are clear risk stratifications within this high risk group that may exclude people with much lower risk for prevalent TB – e.g. those receiving ART for less than 6 months or non-adherent to ART; pregnancy or post-partum; CD4 count less than 350 cells/mm^3, etc., we will start this study with the simple inclusion factor, without riders or sub-clauses. This may change as more data is collected (12, 37, 38).
- **History of prior TB disease in the past 2 years**. Multiple studies show those who have had a prior episode of TB are at very high risk of recurrence especially the first year after first episode (21).
- **Contact of adolescent or adult with TB in the past year**. TB incidence in contacts of a TB case are at greatest risk for TB disease in the first year after exposure (40).

Those facility attendees in high risk groups will be tested not more than six-monthly if they do not have symptoms; if symptomatic at any time additional testing will be done according to current guidelines. Facility attendees that do not fall into any of these high-risk categories will be regarded as moderate-risk, and standard of care TB symptom screen will be performed routinely by PHC clinic staff.

A minimum database on all consented participants will be collected from interviews and the medical record. Medical records are in this case defined as the information that is routinely captured by the site's clinical staff for the management of patients, including but not limited to:

- Prescription records
- NHLS laboratory results
- Clinicians', nurses', and counselors' notes
- Demographic data forms
- TB registers (paper and electronic)

The following fields will be collected from participant interview and subjects' medical records for all objectives: age, gender, HIV-serostatus, antiretroviral therapy status and start date, most recent CD4 count available in clinical record, prior diagnosis/es of TB disease, outcome of TB disease and whether or not the attendee reports a TB contact. The specimen bar code will be obtained for each sputum specimen sent in the intervention arm. A symptom screen will be done on each participant.

Figure 1. Study Process schema

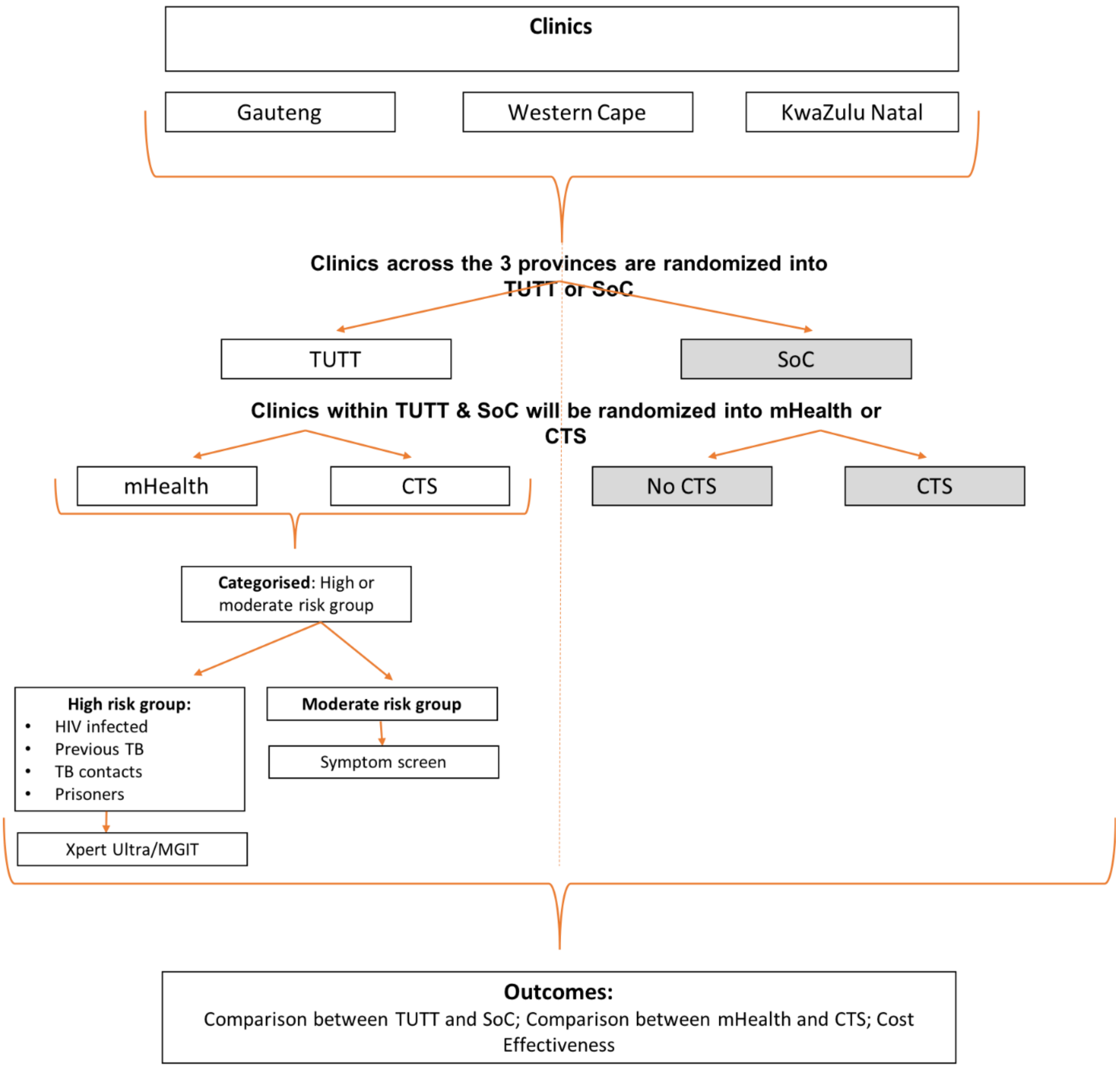

Although actual screening and the processes might vary per clinic depending on the clinic flow, a review of clinic processes suggests patients have long waiting periods to access their medical records, to have vitals measured, to have their medical consultation and the collection of medication at pharmacy. We plan three possible times that patients in the clinic will be reviewed to assess whether they fit into high risk categories Waiting periods, particularly that whilst waiting for clinical consultation (almost two hours) will be used to provide information on TB screening in intervention clinics. The clinic's health promoter will provide information on the study, and request people with a recent TB case at home or recent TB to meet with the fieldworker for TB testing. When patients have received their medical records, these will have a rapid scan to ascertain HIV serostatus. If HIV-infected, they will have sputum collected, preferably before the medical consultation. Additionally, healthcare workers will receive training on TUTT and will be requested to review if the fieldworker might have missed any patients for screening. Later in the day, once the bulk of new patients have arrived at the clinic and have received information, and high risk patients have had sputum collected, and while patients are awaiting their medications, the health

promoter will also review records to ensure that no patient that needs sputum collection for TB screening has been missed. The health promoter will also work in close collaboration with the HIV counseling and testing counsellors, the ARV program and other healthcare workers to ensure that all HIV infected patients receive TB screening every six months.

Moreover, the study will implement quality assurance measures which will include exit interviews and record reviews to ascertain fidelity to protocol in intervention and standard of care clinics.

No TUTT or any other intervention related to TB screening or testing will be implemented at the PHC clinics randomized to the SoC arm. In facilities randomized to TUTT, the health promoter – trained, employed with oversight by the study - will be responsible for identifying high risk patients and ensuring that all identified provide a single spot 4ml sputum specimen will be subjected to Xpert Ultra and MGIT testing after decontamination and sputum homogenization in an NHLS laboratory in each province.

In each facility, where participants have provided sputum for TB investigation, a staff member will be allocated to ensure that results are obtained from the site-specific NHLS laboratory and patients are notified following routine PHC clinic tracing methods. All those newly diagnosed with TB will be initiated on TB treatment.

Healthcare workers and study fieldworkers' views regarding the TUTT intervention

Following the completion of the TUTT intervention, we will invite healthcare workers and study fieldworkers from the TUTT PHC clinics to participate in individual interviews. The aim of the interview is to explore the perceptions and acceptability of the TUTT intervention

Finding Contacts of TB Index Cases

The South African national TB guidelines state that one of the priorities of the TB control programme is to trace people exposed to patients with active TB disease. Contacts of TB cases have a very high prevalence of TB – when universally tested for TB. We will pilot and assess an innovative mHealth intervention aimed to increasing the number of TB contacts who return to the clinic. We understand that the South African Department of Health is about to initiate contact tracing cards – which will be given to diagnosed TB index cases. We therefore plan to test augmenting the contact tracing card with text messages to contacts cell phones. Three different models of contact tracing will be compared and learnt lessons from: mHealth assisted contact tracing, contact tracing cards provided by study staff, and standard of care provided routinely by public sector clinic services. We will ascertain whether contact tracing cards augmented by mHealth messaging increases the total number of contacts screened and more importantly whether it increases the total number of TB cases diagnosed.

mHealth Contact Tracing

Twenty of the PHC clinics in TUTT arm will be randomized to receive our integrated mHealth intervention. Newly diagnosed (patients diagnosed within the last month (30 days)) TB index

cases with pulmonary TB, who are ≥18 years, will be approached, invited to participate in the study, if agreeable will be individually consented. We will offer one of two options for contact tracing: (1) contact tracing cards, (2) The participant will provide numbers to the study staffer which will then be sent by a study device – with no reimbursement.

The content of all contact SMS will include information on TB and will motivate contacts to attend a PHC to be screened. Messages will be designed to follow Fisher and Fisher's Information-Motivation-Behavior Skills (IBM) Model (40). The model works on the premise that, for behavior change to occur, contacts must be informed, be motivated, and then follow the required behavioral strategy. The message will also include an option to request additional information via interactive SMS or telephone call.

Message wording will be depending on prior qualitative research. Prior to the implementation of the sms, a workshop will be held with potential clinic attendees, newly diagnosed TB index cases, and Community Advisory Board members where the content of the message will be assessed to ensure that it is: relevant, informative, motivating, and provides guidance on steps to follow. Feedback from the workshop will be used to refine the content of the TB message.

Contact Tracing provided by Study Staff

Ten of the PHC clinics in the TUTT arm will be randomly allocated to receive the standard contact tracing intervention and all non-intervention clinics will receive the standard of care provided by public sector without any intervention from study staffers. All patients newly diagnosed (patients diagnosed within the last month (30 days)) with TB in the standard contact tracing PHC clinics will be given information on the importance of having their contacts screened for TB and will also be issued with contact tracing slips. The text on the slips will invite any close contacts - individuals that are living or working with the index TB case - to go to their nearest PHC clinic so they can be assessed for TB. The standard contact tracing slips (the size and quality of a standard business slip) will contain a logo of the relevant Provincial Department of Health and will be printed in full colour. There will be no identifiers on the slips. All contacts coming to the PHC clinic in the TUTT arm (with an SMS or contact tracing slip as evidence) will have a sputum specimen taken, irrespective of the presence of symptoms, because they are included in the high-risk groups.

We have already briefed the programming collaborator (Tshimologong) and started development: purpose, process- and content-mapping – identifying possible service providers, specifying hardware required, and working through problems and pitfalls. Once the contract is signed we will enter into a subcontract with Tshimologong. We have contacted Djembe for clinic locator software. We anticipate a beta version of the software will be available four months after the signing of the contract. Implementation of the final working version will be two months thereafter. mHealth clinics will be implemented for a period of approximately six months.

Public Sector Contact Tracing Slips

Contacts in the SoC PHC clinics will receive the Provincial Department of Health contact tracing cards (provided they are available at the time of the study) and if they attend the clinic will be investigated by the non-intervention clinics according to routine care provided at that clinic. By having two sets of CTC clinics (a set which we control the process and a set which is routine), we will actively assess whether there are critical steps required to ensure that CTCs are provided to patients and that they are responded to.

Index patients understanding and acceptability of mHealth component.

A telephonic survey will be conducted by the fieldworker two to eight weeks following the enrolment of the index patient into mHealth, to understand the participant's experience and acceptability of the mHealth component. *Healthcare workers and study fieldworker's perceptions of the CTCs*

Following the completion of the intervention, we will invite healthcare workers and study fieldworker the TUTT PHC clinics to participate in individual, open-ended interviews. The aim of the interviews will be to explore their perceptions and acceptability of the mHealth intervention.

Cost and Cost Effectiveness Measures for Entire Study

We will establish the incremental cost of each intervention strategy (excluding costs purely related to research) as input into assessment of budgetary implications and financial sustainability of the strategies. We will assess both financial and economic costs, with the former to inform planning and budgeting, and the latter to inform economic evaluation and comparisons with other interventions. Costs will be reported by activity and line item. The costing and economic evaluation will be reported from a health service and societal perspective, and will reflect good practice in terms of current economic evaluation Reference Cases. The main relevant incremental costs are expected to include: laboratory tests; staff time administering slips, tests, counselling and treatments (in addition to SoC symptom screening which applies to all subjects); and patient/ contact /carer time. Importantly, we will have to assess the costs of incremental treatment resulting from enhanced case detection.

Costs are expected to be determined through a mix of direct, step-down and ingredients based costing in line with resource and data availability. Staff cost estimates will be based on: the number and proportion of patients actually screened, tested and treated under each strategy; the average duration of each type of patient interaction derived from staff and management estimates; and relevant staff remuneration packages. Staff time estimates will be triangulated with service data, direct observation and time diaries for a sample of services. Prices of lab investigations and drugs will be obtained from standard public-sector tender prices, and extrapolation of radiology costs from other studies, if required. Actual utilization of laboratory tests per facility will be obtained from the NHLS/NICD Data Warehouse, and duration and adherence to drug treatment will be estimated from facility

TB control program statistics. Various overhead costs (e.g. management staff; transport; utilities) will be estimated from accounting records and step-down costing methods.

Possible knock-on effects on utilization of laboratory tests or visits will be assessed if possible, but as extensive individual PHC clinical record review is not anticipated, this may be limited by available routine data collected at each PHC clinic. The principal cost measures for both TUTT vs SoC and mHealth vs standard contact tracing will be: 1) Incremental cost per case detected; and 2) incremental cost per case enrolled on TB treatment. The study will also assess; 3) the incremental cost per additional case completing treatment and 4) incremental cost per case cured, but attribution of such outcomes to the interventions may be more difficult given limited availability of information linked to individual patients. Available costing study resources will be used to prioritize accuracy of major costs before smaller costs are explored in more detail. Where uncertainties exist in relation to certain parameters, sensitivity analyses will be performed. The staff and other capacity requirements for the interventions will also be assessed to inform planning and policy. Furthermore, possible implications of extending the interventions to smaller volume or case load contexts (which tend to have higher unit costs) can be explored through modelling.

Data Management and analysis

In order to ensure that there is high fidelity to the protocol, a study team member (monitoring and evaluations officers, supervised by the monitoring and evaluation coordinator) will visit the intervention clinic every month and non-interventions clinics every two months to observe procedures, monitor collection of specimens and conduct spot exit interviews and exit record reviews.

Study identification codes and linking of records: upon consenting to participate in the study, each subject will be assigned a study identification code.

A logbook linking study identification codes to subjects' names, dates of birth, and clinic file numbers will be kept at each facility in a secure location. The log book will be a separate document from any other study files. Access to the logbook file will be limited to the study team.

Data management and storage: Study specific data will be captured on site into a Research Electronic Data Capture (REDCap) database or similar database. Medical record data for study subjects (e.g. TB test results, treatment details, etc.) will be captured from the study clinics' routine medical records, which include both paper and electronic files.

Statistical Considerations

Power and sample size

To estimate power to detect a 25% difference in TB case rates, we anticipate that study facilities sampled from (that is, those with TB case volumes>20 per month) will have numbers of TB cases diagnosed per calendar month ranging from 20 through 35. Prior data used in a current clinic-based TB case-finding study from a rural setting in the Northern

Province (Vhembe and Waterberg districts) of South Africa indicated high heterogeneity in the number of clinic cases with a coefficient of variation (CV) of 0.5 before stratification. Stratification by historical clinic volume led to a weighted coefficient of variation of 0.29. We will follow a similar approach and use stratified randomization within provinces in order to reduce between-cluster variability and to balance assignment between TUTT and SoC arms, or by other similar macro-level variables that may affect variability.

Table 2: Power calculations for different values of weighted between-cluster CV, and the weighted average case-volume over 14 months.

**Weighted Coefficient of variation**	**Power to detect a 25% difference with 30 facilities per arm over 14 months at alpha level=0.05**
0.2	98%
0.25	90%
0.3	78%
0.35	65%

Randomization

We plan to stratify facilities by province and historical volume size (or other pertinent variable/s) to reduce between-cluster variability and to balance assignment between TUTT and SoC arms. Thus, a total of 60 clinics, will be randomized to either TUTT (n=30) or SoC (n=30). Sub-randomization to CTS and non-CTS arms will be conducted within the main arms for clinics only in order to test for additional yield in TB cases that CTS provide, and to evaluate the programs feasibility and acceptability.

Data analysis plan

The main outcome will be the cluster-level TB-case rate which is the number of index TB cases (excluding CTS contact cases) diagnosed over the study period. The null hypothesis to be tested is that the rate ratio of TUTT: SoC is 1 and that can be evaluated using a stratified t-test. Considering that we will likely have stratified randomization by province and historical volume, it will be more efficient to calculate this cluster-level rate ratio using a Poisson regression adjusting for arm and the stratification factors, with the log of the number of study days as the offset, and an over-dispersion parameter to account for heterogeneity in the facility case rates. We will report the corresponding confidence interval and p-value for the test of the null hypothesis that the rate ratio between the two arms is 1.

A secondary analysis will involve evaluating any potential residual confounding by factors not adjusted for during randomization if relevant data become available. These likely will include the HIV-prevalence for the district (within province) that the facility is located and

the prior year's TB case load. We will first fit a regression model of the facility-level log rates on the stratum factor and HIV-prevalence. The residuals from this regression will then be regressed on study arm to obtain the adjusted risk ratio and its test for significance.

The detail of the analysis of outcome data will be according to the most recent version of the statistical analysis plan - agreed upon between the implementers of the study and the study statistician.

CTS v No CTS nested within TUTT or SoC

The aim of this analysis is to evaluate the impact of contact referral to primary care facilities. Our study will be under-powered to test interaction effects of the CTS by TUTT interventions. Thus we will only investigate the additive effects of these interventions (TUTT+CTS) using a Poisson regression similar to the one described above with TUTT/SoC and CTS/noCTS as main effects. We will also report the following summary statistics which are indicators of acceptability and feasibility of using CTS

1. Additional yield in TB cases produced by CTS contact cases in each main arm: the proportion of the CTS screened and tested contacts who were confirmed newly diagnosed TB cases
2. The contact-screened rate: Those who attend the clinic for TB screening because they self-reported they were in contact with a TB case;
3. The absolute number of TB diagnosed who could be linked to handed-out / returned contact tracing slips; either by direct questioning or by bringing the slip back.

Interim analysis and stopping rules

A key issue raised by reviewers is the length of the study and the funder's requirement that actionable results be made available as soon as they are available. We have therefore assessed various options and propose that the DSMB advise investigators in deciding on continuing the trial to collect more data, or prematurely halting it and disseminating results.

We only specify a priori that we will conduct the first interim analysis using data to the end of month 6 of the study. We do not specify the frequency of the rest of the interim analyses nor the boundary values for our test statistic (i.e. for the test Ho: risk ratio=1) at which the study may be stopped. This is because of the following reasons. To determine the optimal stopping point, we need to calculate the amount of information (the information fraction) obtained at each point of the interim analysis (Hayes and Moulton, 2009). This is often taken as the reciprocal of the variance of the effect estimate. In a cluster randomized trial (CRT), this information fraction depends on the number of incidence cases and the coefficient of variation observed in the data thus far. Furthermore, for CRTs, as the events

occur over time the information fraction decreases. The dynamic interplay between time and the information fraction preclude a prior determination of intervals between interim analyse. We will propose to the DSMB that the first unblinded interim analysis they receive for scrutiny will include the data from the first six months of study data from all facilities included in the trial. Based on the 1st unblinded outcome report provided to the DSMB by the study statistician, the DSMB will make an informed decision about the trial proceeding, Should the DSMB request an interim analysis at four months, the study will acquiesce to this request with the caveat that the at an alpha =0.005, we would have 55% power to detect a difference of 25% between the main study arms. Moreover, to ensure that interim analyses are not inadvertently given too much credence by the DSMB, we propose using the Lan-DeMets alpha spending function approach which proposes a higher threshold for rejecting the null hypothesis using interim analyses that include less data than the final analysis would. (***DeMets and Lan (1994) Interim Analysis: the alpha spending function approach, Statistics in Medicine 13, 1341-1352***) This approach ensures that we keep the total Type I error rate within the pre-specified threshold over all interim analyses tests that are conducted. At each interim analysis, the *observed* value of the coefficient of variation will be used to estimate the information fraction and the test statistic bounds for whether to continue the trial or not using the software package *ldmets* in R. A statistical analysis plan detailing the per protocol and as-treated analyses will be used to guide interim and final outcome analyses. The SAP will be finalized prior to any interim or final analysis being performed.

ETHICAL CONSIDERATIONS

The proposed study will be submitted to the Human Research Ethics Committee (Medical) (HREC) at the University of the Witwatersrand and all the Provincial Departments of Health for approval. Participants in the study will undergo informed consent process to participate in the study. Participants who have a positive TB result will be assessed by clinic staff for treatment decisions. The DSMB requested that an advisory panel be created to assist clinicians in making treatment decisions particularly in those without TB symptoms and in those with a history of prior TB and positive Xpert result.

All clinic attendees that are eligible for mHealth contact tracing will be required to sign an informed consent form indicating their contact choice. Furthermore, all workshop participants and healthcare providers will be required to sign an ICF prior to taking part in the study.

REFERENCES

1. Naidoo P, Theron G, Rangaka MX, Chihota VN, Vaughan L, Brey ZO, et al. The South African Tuberculosis Care Cascade: Estimated Losses and Methodological Challenges. The Journal of infectious diseases. 2017;216(suppl_7):S702-s13.
2. Reid A, Grant A, White R, Dye C, Vynnycky E, Fielding K, et al. Accelerating progress towards tuberculosis elimination: the need for combination treatment and prevention. The international journal of tuberculosis and lung disease. 2015;19(1):5-9.
3. World Health Organisation. Global tuberculosis report 2017. Geneva; 2017.
4. Omar T, Variava E, Moroe E, Billioux A, Chaisson RE, Lebina L, et al. Undiagnosed TB in adults dying at home from natural causes in a high TB burden setting: a post-mortem study. The international journal of tuberculosis and lung disease : the official journal of the International Union against Tuberculosis and Lung Disease. 2015;19(11):1320-5.
5. Cohen T, Murray M, Wallengren K, Alvarez GG, Samuel EY, Wilson D. The prevalence and drug sensitivity of tuberculosis among patients dying in hospital in KwaZulu-Natal, South Africa: a postmortem study. PLoS medicine. 2010;7(6):e1000296.
6. World Health Organisation. Systematic screening for active tuberculosis: principles and recommendations. Geneva; 2013. Report No.: 9241548606.
7. National Department of Health. National Tuberculosis Management Guidelines 2014. Pretoria; 2014.
8. Suthar A, Zachariah R, Harries A. Ending tuberculosis by 2030: can we do it? The international journal of tuberculosis and lung disease. 2016;20(9):1148-54.
9. Getahun H, Kittikraisak W, Heilig CM, Corbett EL, Ayles H, Cain KP, et al. Development of a standardized screening rule for tuberculosis in people living with HIV in resource-constrained settings: individual participant data meta-analysis of observational studies. PLoS medicine. 2011;8(1):e1000391.
10. Rangaka MX, Wilkinson RJ, Glynn JR, Boulle A, van Cutsem G, Goliath R, et al. Effect of antiretroviral therapy on the diagnostic accuracy of symptom screening for intensified tuberculosis case finding in a South African HIV clinic. Clinical infectious diseases : an official publication of the Infectious Diseases Society of America. 2012;55(12):1698-706.
11. Wood R, Middelkoop K, Myer L, Grant AD, Whitelaw A, Lawn SD, et al. Undiagnosed Tuberculosis in a Community with High HIV Prevalence: Implications for Tuberculosis Control. American Journal of Respiratory and Critical Care Medicine. 2007;175(1):87-93.
12. Hoffmann CJ, Variava E, Rakgokong M, Masonoke K, van der Watt M, Chaisson RE, et al. High prevalence of pulmonary tuberculosis but low sensitivity of symptom screening among HIV-infected pregnant women in South Africa. PloS one. 2013;8(4):e62211.
13. McCarthy K, Grant A, Chihota V, Ginindza S, Mvusi L, Churchyard G, et al. What happens after a negative test for tuberculosis? Evaluating adherence to TB diagnostic algorithms in South African Primary Health Clinics. J Acquir Immune Defic Syndr. 2015;71:e119-e26.
14. World Health Organisation. Tuberculosis: Fact Sheet 2017 [updated October 2017. Available from: http://www.who.int/mediacentre/factsheets/fs104/en/.
15. Hanifa Y, Fielding KL, Chihota VN, Adonis L, Charalambous S, Foster N, et al. A clinical scoring system to prioritise investigation for tuberculosis among adults attending HIV clinics in South Africa. PloS one. 2017;12(8):e0181519.
16. Nabukalu D, Klipstein-Grobusch K, Herbst K, Newell M-L. Mortality in women of reproductive age in rural South Africa. Global health action. 2013;6(1):22834.
17. National Department of Health. Saving Mothers 2011-2013: Sixth report on the Confidential Enquiries into Maternal Deaths in South Africa. Pretoria, South Africa; 2014.
18. Shapiro AE, Variava E, Rakgokong MH, Moodley N, Luke B, Salimi S, et al. Community-based targeted case finding for tuberculosis and HIV in household contacts of patients with tuberculosis in South Africa. American Journal of Respiratory and Critical Care Medicine. 2012;185(10):1110-6.
19. Becerra MC, Pachao-Torreblanca IF, Bayona J, Celi R, Shin SS, Kim JY, et al. Expanding tuberculosis case detection by screening household contacts. Public health reports. 2005;120(3):271-7.

20. Guwatudde D, Nakakeeto M, Jones-Lopez E, Maganda A, Chiunda A, Mugerwa R, et al. Tuberculosis in household contacts of infectious cases in Kampala, Uganda. American journal of epidemiology. 2003;158(9):887-98.
21. Panjabi R, Comstock G, Golub J. Recurrent tuberculosis and its risk factors: adequately treated patients are still at high risk. The International Journal of Tuberculosis and Lung Disease. 2007;11(8):828-37.
22. Grobler L, Mehtar S, Dheda K, Adams S, Babatunde S, Walt M, et al. The epidemiology of tuberculosis in health care workers in South Africa: a systematic review. BMC health services research. 2016;16(1):416.
23. McCarthy K, Scott L, Gous N, Tellie M, Venter W, Stevens W, et al. High incidence of latent tuberculous infection among South African health workers: an urgent call for action. The International Journal of Tuberculosis and Lung Disease. 2015;19(6):647-53.
24. Patra J, Jha P, Rehm J, Suraweera W. Tobacco smoking, alcohol drinking, diabetes, low body mass index and the risk of self-reported symptoms of active tuberculosis: individual participant data (IPD) meta-analyses of 72,684 individuals in 14 high tuberculosis burden countries. PloS one. 2014;9(5):e96433.
25. Cois A, Ehrlich R. Problem drinking as a risk factor for tuberculosis: a propensity score matched analysis of a national survey. BMC public health. 2013;13(1):871.
26. Bates MN, Khalakdina A, Pai M, Chang L, Lessa F, Smith KR. Risk of tuberculosis from exposure to tobacco smoke: a systematic review and meta-analysis. Archives of internal medicine. 2007;167(4):335-42.
27. Oni T, Berkowitz N, Kubjane M, Goliath R, Levitt NS, Wilkinson RJ. Trilateral overlap of tuberculosis, diabetes and HIV-1 in a high-burden African setting: implications for TB control. European Respiratory Journal. 2017;50(1):1700004.
28. Balcha T, Skogmar S, Sturegård E, Schön T, Winqvist N, Reepalu A, et al., editors. A clinical scoring algorithm for determination of the risk of Tuberculosis in HIV-infected adults: a cohort study performed at Ethiopian health centers. Open forum infectious diseases; 2014: Oxford University Press.
29. Cox HS, Mbhele S, Mohess N, Whitelaw A, Muller O, Zemanay W, et al. Impact of Xpert MTB/RIF for TB diagnosis in a primary care clinic with high TB and HIV prevalence in South Africa: a pragmatic randomised trial. PLoS medicine. 2014;11(11):e1001760.
30. Calligaro GL, Zijenah LS, Peter JG, Theron G, Buser V, McNerney R, et al. Effect of new tuberculosis diagnostic technologies on community-based intensified case finding: a multicentre randomised controlled trial. The Lancet Infectious Diseases. 2017;17(4):441-50.
31. World Health Organisation. Next-generation Xpert® MTB/RIF Ultra assay recommended by WHO 2017 [Available from: http://who.int/tb/features_archive/Xpert-Ultra/en/.
32. Kerkhoff AD, Barr DA, Schutz C, Burton R, Nicol MP, Lawn SD, et al. Disseminated tuberculosis among hospitalised HIV patients in South Africa: a common condition that can be rapidly diagnosed using urine-based assays. Scientific Reports. 2017;7(1):10931.
33. Peter J, Theron G, Chanda D, Clowes P, Rachow A, Lesosky M, et al. Test characteristics and potential impact of the urine LAM lateral flow assay in HIV-infected outpatients under investigation for TB and able to self-expectorate sputum for diagnostic testing. BMC infectious diseases. 2015;15(1):262.
34. Sun D, Dorman S, Shah M, Manabe YC, Moodley VM, Nicol MP, et al. Cost utility of lateral-flow urine lipoarabinomannan for tuberculosis diagnosis in HIV-infected African adults. The International Journal of Tuberculosis and Lung Disease. 2013;17(4):552-8.
35. Zachariah R, Spielmann MP, Harries AD, Gomani P, Graham SM, Bakali E, et al. Passive versus active tuberculosis case finding and isoniazid preventive therapy among household contacts in a rural district of Malawi. The international journal of tuberculosis and lung disease : the official journal of the International Union against Tuberculosis and Lung Disease. 2003;7(11):1033-9.
36. Massyn N PN, English R, Padarath A, Barron P, Day C,. District Health Barometer 2015/16. Durban, South Africa: Health Systems Trust; 2016.
37. Lawn SD, Badri M, Wood R. Tuberculosis among HIV-infected patients receiving HAART: long term incidence and risk factors in a South African cohort. AIDS (London, England). 2005;19(18):2109-16.

38. Hanifa Y, Fielding KL, Chihota VN, Adonis L, Charalambous S, Karstaedt A, et al. Diagnostic Accuracy of Lateral Flow Urine LAM Assay for TB Screening of Adults with Advanced Immunosuppression Attending Routine HIV Care in South Africa. PLoS One. 2016;11(6):e0156866.
39. Johnstone-Robertson S, Lawn SD, Welte A, Bekker LG, Wood R. Tuberculosis in a South African prison - a transmission modelling analysis. South African medical journal = Suid-Afrikaanse tydskrif vir geneeskunde. 2011;101(11):809-13.
40. Fox GJ, Barry SE, Britton WJ, Marks GB. Contact investigation for tuberculosis: a systematic review and meta-analysis. The European respiratory journal. 2013;41(1):140-56.
