# Supplementary material for: Evaluating systematic targeted universal testing for tuberculosis in primary care clinics of South Africa: A cluster-randomized trial (The TUTT Trial)
Source: PLoS Med. 2023 May 22;20(5):e1004237. doi: 10.1371/journal.pmed.1004237 (PMC10263318; doi:10.1371/journal.pmed.1004237)
Supplement: Attachment — Submitted filename: TUTT Final Revision March 2023 Response to EditorsNM.docx [file pmed.1004237.s006.docx]

Requests from the editors:

GENERAL
Thank you for your detailed and considerate responses to previous editor and reviewer requests, please see below for additional comments which we require that you address in full.

**** We note in your abstract that the recruitment to the study was “halted” in March 2020 (coinciding with pandemic lockdowns). Please include details, in the abstract (and the main manuscript) which clearly states why the trial was halted and whether termination of recruitment at this time point has any implications on sample size and statistical powering (you go some way to doing this in the discussion) ****

We have included more detail in the abstract and refer to the premature “halting” of the study and its implications in the results section (and as you noted in the discussion).

DATA AVAILABILITY
**In accordance with ICMJE requirements, PLOS Medicine requires prospective, public registration of a data sharing plan (as part of mandatory clinical trials registration) for all clinical trials that began enrolment on or after January 1, 2019.  Please include details.**

1. We are keen to share our data and are already in a DTA process with the University of Heidelberg in Germany.
2. We will provide data to people who make a reasonable request for it. Our constraint is that the local IRB requires that a data transfer agreement be in place and that the purpose of the request is reviewed by them.
3. In anticipation of requests, we have already had a generic data transfer agreement (DTA) for this trial approved by our internal legal team.
4. These details should now appear on the publicly available pages of the TUTT Trial in South African National Clinical Trials Register (an online database) website.

TITLE
**Please revise your title according to PLOS Medicine's style. Your title must be nondeclarative and not a question. It should begin with main concept if possible. "Effect of" should be used only if causality can be inferred, i.e., for an RCT. Please place the study design ("A randomized controlled trial," "A retrospective study," "A modelling study," etc.) in the subtitle (ie, after a colon).**

The title has been revised to:

“Evaluating systematic targeted universal testing for tuberculosis in primary care clinics of South Africa; a cluster randomized trial (The TUTT Trial)”

ABSTRACT
**Line 52: “Subjecting data extracted…” instead perhaps?**

This has been cleaned up.

**Line 50: please revise to read “…attendees living with HIV…” please check and amend throughout the manuscript where relevant.**

All are amended to include “living with HIV”

**Please include the primary outcome.**

We have clarified this in the abstract.

AUTHOR SUMMARY
**Thank you for including an author summary.  Please trim your summary to include ideally 2-3 (but no more than 4) single sentence bullet points for each of the questions (Why Was This Study Done?, What Did the Researchers Do and Find? , What Do These Findings Mean?). Bullet points should be objective, brief, succinct, specific, accurate, and avoid technical language.
Reviewing existing published articles here** [**https://journals.plos.org/plosmedicine/**](https://nam02.safelinks.protection.outlook.com/?url=https%3A%2F%2Fjournals.plos.org%2Fplosmedicine%2F&data=05%7C01%7Cbnonyan1%40jhu.edu%7C531743dd03ae4ce824c208db1f0ef175%7C9fa4f438b1e6473b803f86f8aedf0dec%7C0%7C0%7C638137921606504760%7CUnknown%7CTWFpbGZsb3d8eyJWIjoiMC4wLjAwMDAiLCJQIjoiV2luMzIiLCJBTiI6Ik1haWwiLCJXVCI6Mn0%3D%7C3000%7C%7C%7C&sdata=uyqk6ucPYZNGcw2QdgQ%2FkibAhLlFAWag27r%2FAheKpuE%3D&reserved=0) **on our website may be helpful to you.**

We have attempted to respond to this. We have trimmed the number of bullet points, and hopefully made them pithy, objective, and with less technical language.

INTRODUCTION
**Line 178: as above please amend to read as follows, “…in people living with HIV…”.  Please check and amend throughout the manuscript where relevant.**

All are amended to include “living with HIV”

METHODS and RESULTS
**PLOS Medicine requires that all trials be prospectively registered in one of registries recognized by WHO. Please provide information on study registration in the Methods section.**

We registered the trial with the South African National Trials Registry (<https://sanctr.samrc.ac.za/Default.aspx>) at the time of submission of the trial to the local IRB.  Like clinicaltrials.gov the South African National Clinical Trials registry is not on the list of registries included on the WHO website but the WHO webpage does include a link to the South African Department of Health.  That link suggests that the SANCTR is “ in accordance with” the WHO trial platform (see page 38  [https://www.witshealth.co.za/Portals/0/SA%20GCP%20GUIDELINES%202006.pdf](https://nam02.safelinks.protection.outlook.com/?url=https%3A%2F%2Fwww.witshealth.co.za%2FPortals%2F0%2FSA%2520GCP%2520GUIDELINES%25202006.pdf&data=05%7C01%7Cbnonyan1%40jhu.edu%7C531743dd03ae4ce824c208db1f0ef175%7C9fa4f438b1e6473b803f86f8aedf0dec%7C0%7C0%7C638137921606504760%7CUnknown%7CTWFpbGZsb3d8eyJWIjoiMC4wLjAwMDAiLCJQIjoiV2luMzIiLCJBTiI6Ik1haWwiLCJXVCI6Mn0%3D%7C3000%7C%7C%7C&sdata=h3xz0IYlBA%2BONgM12Guic43z3yOJWHzNmIGU6eVEAkc%3D&reserved=0)).

Please amend statistical reporting for consistency.  For example, line 450 reads, “…1.14 (95% CI 0.94, 1.38, p=0.46)” but at line 457, “…1.19 (0.99, 1.44) (p=0.05).” Please revise throughout for consistency. We suggest adopting the former style of presentation.

**Done**

**Line 269: Please include whether consent obtained was written or oral.**

It was written informed consent; we have amended: “Fieldworkers received trial-specific training that included: approaching and obtaining individual written consent from participants; …..”

PARTICIPANT CHARACTERISTICS
**Thank you for including details of the clinics include in your trial.
Throughout, specific individual patient characteristics are documented to be required for inclusion into the study, including “high risk” status and the reasons for the risk. Further you specifically document individual consent to participate in the study.  For these reasons and to help facilitate transparency of data reporting we request that you please include the characteristics of the consenting participants.**

We have included a brief summary but in the text refer the reader to this trial’s related manuscript recently published in CID that has a detailed description of the individual participants we consented and recruited but not the trial outcomes. The CID paper should be read in conjunction with the manuscript under review. Please see table 1 in our CID paper: https://academic.oup.com/cid/advancearticle/doi/10.1093/cid/ciac965/6969441?login=false.

FIGURES
**Figure 3 – when reporting p values, please include numerical values instead of using asterisks as indicators**

The majority of the p-values listed in Figure 3 are small and output as “0” by the analysis package. These are typically reported as p<0.001 and this we indicated in the caption. Adding this additional text (“p<=0.001”) within the figure interferes with the readability of the key data in the figure. We therefore, prefer to keep this in the caption.

STATISTICAL ANALYSIS PLAN
**In your rebuttal, you state that no statistical analysis plan was made but in the abstract, you write “pre-specified difference in-differences analyses” and in the manuscript submission form you state “the local IRB has acknowledged both the planned analysis and…” which would suggest you had a pre-specified analysis plan please clarify/amend to include the statistical analysis plan that you refer to.**

We have clarified this.  Indeed we had prepared formal statistical analysis plan which was presented to the DSMB prior to us starting the analysis. We have corrected the manuscript to reflect this.

DISCUSSION
**Please remove the declarations of interest statement and the data sharing statements from the end of the discussion and include only in the manuscript submission form.**

Done

REFERENCES
**For in-text reference callouts, please ensure a) an absence of spaces between citations and b) a space preceding the opening parenthesis. For example, line 147 “…cases (10 million) [1,2]…”. Please check and amend throughout.**

Done

Comments from the reviewers:

Reviewer #1: My comments have been satisfactorily addressed.

**Thank you!**


Reviewer #3: I think the authors have adequately answered/addressed my questions and questions from the other reviewers. I think the paper is acceptable in its current form. Congratulations!

**Thank you!!!**
