# Supplementary material for: Evaluating systematic targeted universal testing for tuberculosis in primary care clinics of South Africa: A cluster-randomized trial (The TUTT Trial)
Source: PLoS Med. 2023 May 22;20(5):e1004237. doi: 10.1371/journal.pmed.1004237 (PMC10263318; doi:10.1371/journal.pmed.1004237)
Supplement: Attachment — Submitted filename: Response to Editors April 2023.docx [file pmed.1004237.s007.docx]

The Editors

PLOS Medicine

19 April 2023

**Evaluating systematic targeted universal testing for tuberculosis in primary care clinics of South Africa; a cluster randomized trial (The TUTT Trial)" (PMEDICINE-D-22-03274R3). Remaining Issues**

We provide our responses to each of these recommended revisions below.

1. **Thank you for revising the author summary. Please see below for recommended revisions, as per our understanding, to improve accessibility and brevity. Please check carefully to ensure accuracy. Where [….] are presented please fill in the blanks**

We have made minor editing (yellow highlights) as well as filling in the blanks.

Why Was This Study Done? 
o TB diagnosis is made by laboratory sputum testing usually prompted by the presence of at least one symptom of TB (cough, fever or night sweats, loss of weight). 
o The World Health Organization promotes screening of entire groups of people at high risk of TB irrespective of symptoms.  In South Africa high risk groups include people living with HIV; those who report being a close contact of someone with TB; and those who have had a recent episode of TB.
o There is little data on the usefulness of diagnostic assays as screening tests. Our trial assessed if targeted sputum testing of people at high risk without TB symptoms helped to identify undiagnosed TB.

What did the researchers do and find?
o 62 large primary care clinics in South Africa were randomised to either our intervention -TB testing of sputum of all people in high-risk groups, or our control - to continue symptom directed testing for TB (the standard of care). 
o The total number of TB cases diagnosed each month in all clinics was recorded. Comparisons were made between intervention and control clinics. 
o In trial intervention clinics 6777 people were diagnosed with TB, an average of 20.7 patients per clinic month versus 6750 in control clinics, an average of 18.8 patients with TB per clinic month. 
o After adjusting for clustering, province, and the strata of average number of TB patients diagnosed per month in the last quarter of 2017, intervention clinics diagnosed 14% more patients with TB than control clinics but this did not reach statistical significance.  Secondary analyses, including data from the year prior to the intervention demonstrated a statistically significant increase in TB diagnoses per month, reported as a 17% relative increase.

What do these findings mean?
o A strategy targeting high risk groups for universal testing for TB (TUTT) may help to improve diagnostic rates of TB in areas where prevalence is high.
o Universal testing strategies could be be implemented in low-resource settings provided costs of testing were addressed; and other locally relevant high risk groups for TB could be targeted.

2. PARTICIPANT CHARACTERISTICS
**We previously said the following:
“Thank you for including details of the clinics include in your trial.
Throughout, specific individual patient characteristics are documented to be required for inclusion into the study, including “high risk” status and the reasons for the risk. Further you specifically document individual consent to participate in the study. For these reasons and to help facilitate transparency of data reporting we request that you please include the characteristics of the consenting participants.”

Author response: “We have included a brief summary but in the text refer the reader to this trial’s related manuscript recently published in CID that has a detailed description of the individual participants we consented and recruited but not the trial outcomes.  The CID paper should be read in conjunction with the manuscript under review.  Please see table 1 in our CID paper:** [**https://academic.oup.com/cid/advancearticle/doi/10.1093/cid/ciac965/6969441?login=false.”**](https://academic.oup.com/cid/advancearticle/doi/10.1093/cid/ciac965/6969441?login=false.”)

**We were unfortunately unable to access the manuscript via the link above but the editorial team agree that it would be unfair to expect the reader to refer to another paper for this information.
Please include a table of baseline characteristics of your study population within either the main manuscript or as supporting information. Please ensure that in the manuscript text you direct the reader to where this can be found. This is a prerequisite for publication.**

1. It appears the manuscript may have moved from advance article status to published article status. Now available at [10.1093/cid/ciac965](https://doi.org/10.1093/cid/ciac965)
2. We have included a supplementary table that has the participant characteristics and refer to it in the text.

3. FIGURE 3
**The formatting of this figure is inconsistent with PLOS Medicine’s formatting requirements and requires revision.
Numerical values should be present as 0.943, as opposed to .943. Please revise.
Please ensure that the solid dividing lines of your plots do not strike through the plot titles (fig 3b).  Titles/headers should be placed above the plots.
Asterisks are not permitted please revise to include p values. Please report as p<0.001 and where higher as p=0.002, for example. Please do not report as p<.001 or as p=.002
In figure 3a there are both asterisks and complete values (2nd row ‘medium’). At line 4 (‘GP’) dots and lines overlap numerical values and text – please revise.
Please ensure that all abbreviations are defined (IRR, CI, GP, KZN, WC) either in an appropriate footnote or in the figure caption.**

We now include a figure in the online submission, formatted as requested above.

4. REFERENCES
**For all web references please include an access date**

Done

5. CONSORT CHECKLIST
**Please refer to section and paragraph numbers as opposed to page/line numbers as these often change at the time of publication.**
**SOCIAL MEDIA
To help us extend the reach of your research, if not already done so, please provide any Twitter handle(s) that would be appropriate to tag, including your own, your co-authors’, your institution, funder, or lab. Please detail any handles you wish to be included when we tweet this paper, in the manuscript submission form when you re-submit the manuscript.**

We will do so
